# Enhancing Food Image Recognition by Multi-Level Fusion and the Attention Mechanism

**DOI:** 10.3390/foods14030461

**Published:** 2025-01-31

**Authors:** Zengzheng Chen, Jianxin Wang, Yeru Wang

**Affiliations:** 1 School of Information, Beijing Forestry University, Beijing 100083, China; zengzheng@bjfu.edu.cn; 2 Risk Assessment Division 1, China National Center for Food Safety Risk Assessment, Beijing 100022, China

**Keywords:** convolutional neural network, food recognition, self-attention mechanism, feature fusion, food science and technology

## Abstract

As a pivotal area of research in the field of computer vision, the technology for food identification has become indispensable across diverse domains including dietary nutrition monitoring, intelligent service provision in restaurants, and ensuring quality control within the food industry. However, recognizing food images falls within the domain of Fine-Grained Visual Classification (FGVC), which presents challenges such as inter-class similarity, intra-class variability, and the complexity of capturing intricate local features. Researchers have primarily focused on deep information in deep convolutional neural networks for fine-grained visual classification, often neglecting shallow and detailed information. Taking these factors into account, we propose a Multi-level Attention Feature Fusion Network (MAF-Net). Specifically, we use feature maps generated by the Convolutional Neural Networks (CNNs) backbone network at different stages as inputs. We apply a self-attention mechanism to identify local features on these feature maps and then stack them together. The feature vectors obtained through the attention mechanism are then integrated with the original input to enhance data augmentation. Simultaneously, to capture as many local features as possible, we encourage multi-scale features to concentrate on distinct local regions at each stage by maximizing the Kullback-Leibler Divergence (KL-divergence) between the different stages. Additionally, we present a novel approach called subclass center loss (SCloss) to implement label smoothing, minimize intra-class feature distribution differences, and enhance the model’s generalization capability. Experiments conducted on three food image datasets—CETH Food-101, Vireo Food-172, and UEC Food-100—demonstrated the superiority of the proposed model. The model achieved Top-1 accuracies of 90.22%, 89.86%, and 90.61% on CETH Food-101, Vireo Food-172, and UEC Food-100, respectively. Notably, our method not only outperformed other methods in terms of the Top-5 accuracy of Vireo Food-172 but also achieved the highest performance in the Top-1 accuracies of UEC Food-100.

## 1. Introduction

Food recognition is significant and widely applied in practical use [1]. It can be used in food delivery and personal consumption processes to help detect the types and quantities of food to prevent allergic reactions [2,3]; meanwhile, food recognition can also be applied in diet control. It plays a role in tracking and quantifying nutrient intake [4] to assist people in achieving balanced diets and preventing obesity and chronic diseases [5]. Moreover, food image recognition holds significant theoretical value [6,7,8,9] as an essential part of fine-grained image classification research. Due to the aforementioned factors, there has been a significant focus on food recognition within the domain of computer vision [10,11,12,13].

In food recognition, we refer to a large category of food as a supercategory, such as fruits, vegetables, pasta, and meat. Foods with minor differences within the same supercategory are referred to as subclasses [14]. For example, the supercategory of beef can be subdivided into different subclasses, such as curry beef rice and braised beef rice. Accurately distinguishing these visually similar but different subclasses is the core task of FGVC [15]. However, the current classification accuracy still requires enhancement to enable large-scale practical applications. This can largely be attributed to the challenges faced by FGVC, specifically inter-class similarity and intra-class variability, along with a major obstacle in food image classification: the difficulty in capturing complex local features.

Inter-class similarity and intra-class variability are inherent of FGVC tasks. Identifying attention regions is crucial for addressing these effects. However, in food recognition, defining and locating attention regions is challenging, mainly because attention information varies from object to object. This problem arises from the lack of spatial uniformity in food images, i.e., due to the significant differences in the shape, color, and form of food, there are no geometric structures or patterns that can quickly identify differences. Furthermore, food recognition encounters specific challenges that are not typically encountered in general object recognition tasks. These challenges include the presence of non-rigid objects [3] and the lack of distinct spatial arrangements and key points. Additionally, images depicting the same type of food can exhibit significant variations due to differences in cooking methods and storage durations. Conversely, images from different food categories may share certain similarities. Consequently, traditional machine learning classification models face difficulties when attempting to learn and distinguish the spatial and color features present in these food images [16].

As CNNs have received widespread attention, current research focuses on developing FGVC algorithms based on the latest CNN architectures (such as ResNet [17], TResNet [18], SENet-154 [19], etc.). Shallow networks primarily process low-level features like texture and shape variations, while deeper layers in the network encode information to capture more abstract and semantically relevant features, such as recognizing chicken chunks, poached eggs, and vegetable leaves. Existing advanced CNN designs usually add classifiers at deeper layers of the network, which allows the inheritance of features captured by shallow layers through forward propagation and provides higher accuracy. For example, Fakhrou et al., merged the InceptionV3 and DenseNet201 models and evaluated the outcomes using an average voting decision approach [20]. Similarly, McAllister et al. [21] utilized ResNet-152 and GoogleNet Inception, two distinct pre-trained CNN models, to extract features from images of food, employing five common classifiers to categorize different food types.

**Figure 1:**
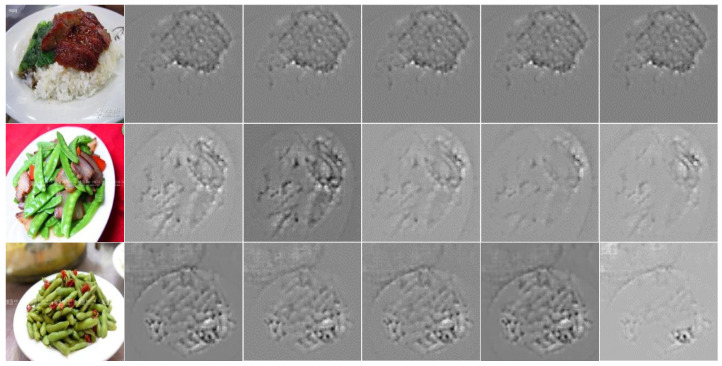
Visualization results from guided backpropagation (GB) [22], implemented using AlexNet [23], trained on Vireo Food172 [24]. The deeper CNN layers concentrate on semantically significant regions while abstracting low-level information acquired by shallow layers. However, this depth in CNN layers may result in the loss of certain details.

However, we believe that even the fundamental information learned by shallow networks can offer effective clues for FGVC tasks. For example, as shown in Figure 1, for the first row of char siu rice, the green vegetables, and char siu meat are usually important clues for recognizing it. These deepest layers mainly focus on this discriminative and semantically meaningful information. However, the deepest layers miss some basic but potentially useful details, such as the texture of rice (e.g., the granular texture of rice compared to the form of pasta is crucial for distinguishing between rice dishes and pasta). These details are abstracted into semantic information during shallow layer learning, resulting in a loss of many details.

Based on the preceding discussion, we propose the MAF-Net. Our method utilizes CNNs as the backbone network (specifically, ResNet50 [25] is used in this paper). It produces feature maps that encapsulate multi-scale information of the input image at various stages of the network. These feature maps capture both low-level details and high-level global abstract semantic information. Unlike traditional CNNs that use only the deepest layer for prediction, MAF-Net allows multiple layers to provide classification labels. At the same time, we introduce a self-attention mechanism that uses the feature maps from each level to generate heatmaps, thereby more accurately identifying and focusing on critical local features to enhance them within a single feature vector. Furthermore, these local features are integrated and overlaid, forming a more comprehensive and rich representation, thereby improving the model’s recognition performance. Additionally, we fuse the original input with the attention maps to augment original data when generating new images.

MAF-Net adopts a multi-stage training method to achieve its objectives. First, we train the deepest level of the network, which can accurately distinguish the superclasses of food. Next, we iteratively and progressively utilize prior knowledge to obtain more accurate results (e.g., improved top-5 accuracy on the Vireo Food-172 dataset). In the meantime, updates to the deepest layer parameters will influence the optimization of other stages and their corresponding parameter settings, which in turn helps to refine the network’s overall performance in achieving its objectives. These attention regions demonstrate the enhancement processing effect of the original input data in subsequent stages, and fusion operations are performed at all stages to differentiate different subclasses.

To improve the model’s capability to capture features at different network levels, we introduce a KL-divergence maximization strategy between different stages, which aims to make different stages focus on different local areas of the image. In addition to the aforementioned methods, we also innovatively propose a new loss function called Subclass Center Loss (SCloss). The main objective of this loss function is to incorporate label smoothing and reduce the distribution disparities among features belonging to the same class. By minimizing the variability of intra-class features, we can greatly improve the model’s capacity for generalization and enable it to maintain a high level of accuracy and stability when encountering novel data. Therefore, the model successfully achieves feature enhancement at the same level, between different levels, across the entire category.

In summary, this paper presents the Multi-level Attention Feature Fusion Network (MAF-Net), designed to enhance fine-grained food image recognition. MAF-Net combines multi-level feature maps from a CNN backbone with a self-attention mechanism to more effectively capture crucial local features. We also introduce a KL-divergence maximization strategy and Subclass Center Loss (SCloss) to improve multi-scale feature representation and generalization, leading to significantly higher recognition accuracy.

The structure of the paper is organized as follows: Section 2 reviews related work; Section 3 details the design and implementation of MAF-Net; Section 4 describes the experimental settings and results, validating the effectiveness of our proposed method through comprehensive experiments. Finally, Section 5 summarizes the main contributions of this paper and outlines future research directions.

## 2. Related Work

This section reviews pertinent research in the domains of fine-grained visual classification (FGVC) and food recognition. Initially, we examine FGVC approaches that leverage local features and attention mechanisms, highlighting their advancements. Subsequently, we explore deep learning methodologies and their applications in food recognition. Finally, we summarize the limitations of existing studies and present the improved methods introduced in this paper.

### 2.1. Fine-Grained Visual Classification

Fine-grained visual categorization (FGVC) faces a significant challenge due to the small differences between classes and the large variability within each class. Early methods, such as SPDA-CNN [26] and Mask-CNN [27], focused on detecting local regions of interest to help distinguish fine-grained objects. However, these approaches required precise supervised annotations, like part labels or bounding boxes, which limited their real-world applicability. Defining common parts for certain object classes, such as food dishes, can be especially challenging [28].

In recent years, FGVC has moved toward weakly supervised learning, where only image-level class labels are used, without the need for detailed annotations. Attention mechanisms have emerged as a powerful tool for this task, as they can automatically focus on relevant parts of the image to improve classification performance. For example, models like Cross-X [29], ACNet [30], and CAL [31] have successfully employed attention mechanisms to enhance their fine-grained classification capabilities. Moreover, recent work, including TTransFG [32] and CAMF [33], demonstrates that integrating transformers with attention mechanisms can further improve accuracy. Additionally, Wang et al. [34] proposed a method that uses feature map channel information to create a heterogeneous branch structure, which separates global and local features for more efficient processing. Chen et al. [35] presented a technique to enhance classification accuracy by distorting and reconstructing images. This model can effectively learn target structures and local discriminative regions to improve accuracy significantly.

The methods above mainly focus on the local information contained in images for FGVC tasks; however, when dealing with food images, the local information used to distinguish food categories may be more than one kind. To address this, we maximize the KL-divergence across various CNN stages, encouraging multi-scale features to concentrate on distinct local regions at each stage, thereby enhancing the capture of local features.

### 2.2. Food Recognition

This field includes sub-domains such as food recognition [12,13], food detection [36], segmentation [37], and retrieval and generation [38,39]. The identification of food is imperative, serving as a foundational prerequisite for more intricate endeavors such as exploration, recipe development, and dietary intake monitoring.

In earlier research, handcrafted features like Histogram of Oriented Gradients (HOG) [40] were widely used for image classification and detection. HOG effectively captures low-level features such as gradients and edges, making it useful in tasks like pedestrian detection. However, it struggles with high-level semantic information due to its reliance on predefined features. In contrast, Convolutional Neural Networks (CNNs) learn both low- and high-level features through end-to-end training, making them more suitable for complex tasks like fine-grained food recognition. This shift to deep learning has greatly improved the accuracy and robustness of food recognition systems, enabling more advanced applications.

As one of the focal points of research in computer vision and multimedia, food recognition has always been a fundamental task in food computing. Early research work [25,41,42] has adopted manual feature design methods, such as using SIFT-based bag-of-words features [41] and local binary pattern features [42] for recognition. However, with the rapid advancement of deep learning technology, CNNs have emerged as the predominant approach for food recognition tasks [43,44]. For example, Kagaya et al. used AlexNet to extract visual features for food recognition and detection tasks [43]. Subsequently, some researchers have proposed methods focusing on the fine-grained features of food [12,45]. For instance, Qiu et al. [45] proposed a dual-network approach that combines global and local feature representations to enhance classification accuracy.

Various methods have been proposed for health-related tasks to suit different application scenarios. Ciocca et al. designed datasets and methods specifically tailored for CNN applications in dietary monitoring [46]. Another noteworthy approach is the system developed by Bossard et al. [47], which used deep learning technology to monitor daily dietary energy expenditure and captured and estimated the beverage content through smartphone cameras.

Considering the characteristics of food images, this paper focuses on the features output by CNNs at different levels. It incorporates shallow information such as texture as part of the discrimination. Additionally, we propose an evaluating method called subclass center loss (SCloss) to reduce intra-class feature distribution differences and enhance the model’s generalization ability.

## 3. Approach

**Figure 2:**
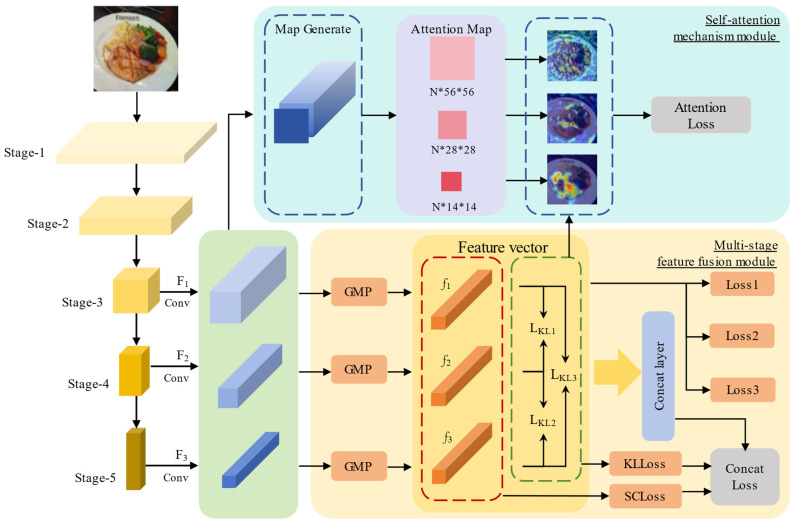
The framework of MAF-Net. It includes a multi-stage feature fusion module and a self-attention mechanism module.

In this section, we introduce the MAF-Net, as illustrated in Figure 2. The backbone of this network can utilize advanced CNN architectures, such as ResNet50 [25] and Res2NeXt50 [48]. We extract feature vectors at different stages to identify local features at various levels. A self-attention mechanism is employed at each stage to capture the correlations between different local features, thereby improving the final classification results. To enhance the model’s generalization ability and reduce intra-class feature distribution differences, we propose a new method called subclass center loss (SCloss). Finally, the complementary outputs from each stage are combined for food classification prediction during inference.

### 3.1. Multi-Level Feature Learning

In food classification, we define a broad grouping of foods as a super-class, while closely related or similar foods within the same super-class are categorized into sub-classes. Even though food image recognition involves fine-grained visual tasks, the deeper layers of a CNN are capable of effectively recognizing the majority of images. However, within the same super-class, there are highly similar features between different sub-classes; therefore, we need to use more complex methods to focus on finer-grained local features.

The diagram below illustrates the architecture of MAF-Net. The backbone network employed in this study is ResNet50, which accepts a batch of images sized 448 × 448 pixels as input and produces three feature maps at Stage-3, Stage-4, and Stage-5. We refer to the feature maps generated at these stages as $F_{1}$, $F_{2}$, and $F_{3}$. According to the ResNet-50 network structure, $F_{1}\in {\mathbb{R}}^{B\times 512\times 56\times 56}$, $F_{2}\in {\mathbb{R}}^{B\times 1024\times 28\times 28}$, and $F_{3}\in {\mathbb{R}}^{B\times 2048\times 14\times 14}$, where B is the number of images in each batch. We scale the size of the feature map $F_{n}\;(n=1,2,3)$ to obtain

(1) $$F_{n}^{a}={\mathrm{Conv}}_{n}\left(F_{n}\right),$$

where $Conv_{n}\;(n=1,2,3)$ is a convolution kernel of different sizes, yielding $F_{1}^{a}\in {\mathbb{R}}^{B\times 1024\times 56\times 56}$, $F_{2}^{a}\in {\mathbb{R}}^{B\times 1024\times 28\times 28}$, and $F_{3}^{a}\in {\mathbb{R}}^{B\times 1024\times 14\times 14}$.

Researchers typically use data from $F_{3}^{a}$ at the final layer of a CNN for improvements. Though this method effectively distinguishes super-classes of food images, it has limited accuracy for sub-class discrimination. We use multi-level features $F_{n}^{a}(n=1,2,3)$ to improve recognition accuracy. Specifically, for the output $F_{n}^{a}$ from each stage, we begin by employing a global max pooling (GMP) layer to extract their feature vectors, as follows:

(2) $$f_{n}=\mathrm{GMP}\left(F_{n}^{a}\right),$$

where $F_{n}^{a}(n=1,2,3)$ represents the result of global max pooling on $F_{n}^{a}$, resulting in a 1024-dimension feature $f_{n}\in {\mathbb{R}}^{B\times 1024\times 1\times 1}$. For the obtained result $f_{n}$, we use feature extraction methods and a self-attention mechanism for learning. In the next subsection, we will explain how to leverage the self-attention mechanism to enhance the classification performance of the same network layer. First, we will explain how to use it as a basis to extract features and learn local areas. This process can be roughly expressed by the subsequent formula:

(3) $$f_{n}^{\prime }=f_{Res}(f_{n},(B,1024)),$$

(4) $$f_{n}^{a}=f_{Clas}(f_{Feat}(f_{n}^{\prime })),$$

where $f_{Res}$ is the projection of $f_{n}$ into B×1024, $f_{n}^{\prime }\in {\mathbb{R}}^{B\times 1024}\;(n=1,2,3)$. Next, $f_{n}^{\prime }$ is input into the $f_{Feat}$ function, which performs feature extraction and transformation on the input through batch normalization, linear transformation (from 1024 dimensions to 512 dimensions), and the ELU activation function. The result is then input into the $f_{Clas}$ function, setting the output channels to the quantity of food categories to obtain the result $f_{n}^{a}(n=1,2,3)$ for the final classification decision, thus obtaining the classification loss $L_{n}$ for each stage. Finally, we combine $f_{n}^{a}$ to obtain the final representation $f_{Con}$ at the Concat stage, thus obtaining the loss $L_{Con}$ at the Concat stage.

However, simply using feature vectors generated at different stages from the same original input cannot obtain diversified fine-grained features because the feature information obtained at different stages may focus on similar regions and cannot capture richer local information. To address this, we combine the feature vectors with the original input image at each stage. Specifically, we first apply global max pooling to extract feature vectors from each stage and then merge them with the original input image to form an augmented image pool. This augmented image pool is used in subsequent training to promote diversity in the learned features and improve the model’s ability to capture detailed local features. Next, if features are extracted from the N stages of the backbone network, then from the 2nd to the N-th round of training, we randomly select an input from a pool of images composed of the original input and attention regions extracted from outside that stage and optimize the functional differences in the KL-divergence between them during training to increase their diversity, thus better solving the above problem. The specific training process of MAF-Net will be detailed in the third section of this chapter.

By augmenting the KL-divergence between features at various stages, we promote their focused local features to concentrate on regions that are as distinct as possible at each stage to capture more diverse details. Additionally, this process ensures that the feature vectors at different stages are integrated with the original input image. By randomly selecting inputs from an image pool consisting of both the original input and attention-enhanced regions, we increase the diversity of the features learned by the network and ensure that they focus on different regions, improving classification performance. KL-divergence is used to quantify the similarity between two distributions. By maximizing KL-divergence, we promote greater distinction among features at various stages, allowing the network to capture more nuanced and diverse visual features for recognition.

(5) $$D_{KL}(f_{i}∥f_{j})=\sum_{k}f_{i}(k)\log\left(\frac{f_{i}(k)}{f_{j}(k)}\right),$$

(6) $$L_{KL}=\sum_{1\leq i<j\leq n}D_{KL}(f_{i}∥f_{j}),$$

where $f_{i}$ and $f_{j}$ represent the feature vectors of outputs at different stages, k represents the k-th element of the feature vector, and $f_{i}(k)$ represents the value of the feature vector $f_{i}$ at the k-th position. The method of calculating the loss value $L_{KL}$ using KL-divergence is shown in Equation (6). It achieves this goal by summing the KL-divergence between feature vectors at each stage.

### 3.2. Self-Attention Mechanism

Unlike other tasks that require fine-grained analysis, food images lack static semantic information. Many existing approaches for food recognition primarily focus on directly extracting distinctive features [45], often neglecting the interrelationships among local features within the same network layer. Therefore, we propose the utilization of a self-attention mechanism to discern the interrelationships among distinct regional characteristics. Additionally, this approach allows us to merge the attention map with the original image, resulting in an enhanced feature representation. Consequently, we employ a self-attention mechanism to identify and understand the relationships among various local features. Specifically, we use feature maps $f_{n}$ extracted from different stages of ResNet50, generate query, key, and value features through convolution operations, calculate attention weights, and generate new feature representations to capture essential features better, as follows:

(7) $$q^{\left(i\right)}=\mathrm{Conv}(f_{n}^{\left(i\right)}),$$

(8) $$k^{\left(j\right)},v^{\left(j\right)}=\mathrm{Conv}(f_{n}^{\left(j\right)}),$$

(9) $$S_{i,j}=\mathrm{Softmax}\left(\frac{q^{\left(i\right)}k^{(j)T}}{\sqrt{d_{k^{\left(j\right)}}}}\right),$$

(10) $${\hat{f}}_{i,j}=S_{i,j}v^{\left(j\right)},$$

where $f_{n}^{\left(i\right)}$ and $f_{n}^{\left(j\right)}$ are the i-th and j-th feature positions in $f_{n}$, respectively. Through convolution operations (Conv), we generate query ($q^{\left(i\right)}$), key ($k^{\left(j\right)}$), and value features. Furthermore, we calculate the attention weight $S_{i,j}$ to illustrate the similarity between $q^{\left(i\right)}$ and $k^{\left(j\right)}$. Finally, using the obtained attention weights $S_{i,j}$, we generate new feature representations ${\hat{f}}_{i,j}$. Through this strategy, our model can achieve interactions between different features within the same network layer and obtain the enhanced feature vector $f_{n}^{att}$.

Furthermore, we use $f_{n}^{att}$ to locate and amplify key areas in the image to combine it with the original input x to generate a new image. Specifically, first, we normalize the attention map $f_{n}^{att}$, mapping its values to the [0, 1] interval, as follows:

(11) $${\hat{f}}_{n}^{att}=\frac{f_{n}^{att}-\min(f_{n}^{att})}{\max(f_{n}^{att})-\min(f_{n}^{att})}.$$

Then, we apply a threshold θ to filter high-attention regions in the attention map, as follows:

(12) $$R=\left\{\begin{array}{ll} 1 & \mathrm{if}\;{\hat{f}}_{n}^{att}>\theta \\ 0 & \mathrm{if}\;0<{\hat{f}}_{n}^{att}\leq \theta \end{array}\right..$$

Next, we determine the boundaries of the high-attention region R in the attention map. For the first step, find the indices of all non-zero elements in R, which correspond to high-attention regions in the attention map. Then, we calculate the minimum and maximum values of these indices to establish the boundaries of the cropping area. After determining the cropping area, we crop the original image x according to these boundaries. The cropped image contains the high-attention regions identified in the attention map with the surrounding background. Then, we use bilinear interpolation to resize the cropped image back to the original image size, thus obtaining the new image $x_{n}^{att}$. $x_{n}^{att}$ is used together with the original input to form an image pool to achieve data augmentation effects in subsequent multi-level training. Detailed content and the MAF-Net training process will be introduced in the third section of this chapter.

After obtaining the attention-enhanced feature vectors $f_{n}^{att}$ from each stage, we integrate them to form the final representation $f_{Con}^{att}$ at the Concat stage of the attention phase, as follows:

(13) $$f_{Con}^{att}=\mathrm{Concat}\left(f_{1}^{att},f_{2}^{att},f_{3}^{att}\right).$$

Using $f_{n}^{att}$ and $f_{Con}^{att}$, we can generate classification results ${\left(f_{n}^{att}\right)}^{a}$ and ${\left(f_{Con}^{att}\right)}^{a}$ with self-attention mechanism feature vectors.

### 3.3. Training and Feature Fusion of MAF-Net

MAF-Net’s training is not achieved overnight. We conduct multiple training sessions to gradually combine the local features learned from feature extraction with the self-attention mechanism, aiming to continuously improve the network’s image classification performance.

Specifically, if we choose N layers of the CNN for feature extraction, MAF-Net requires N+2 steps to complete the training, as shown in Algorithm 1. First, train the deepest network layer using the original image x. Next, in steps 2 to N, randomly select an input from the image pool composed of the original input x and the new attention-enhanced images $x_{n}^{att}$ obtained from stages other than the current one. The deep network can combine the knowledge acquired by the shallow network to more accurately represent the underlying classification tasks. By incorporating the attention mechanism into the deep network, the shallow network is enabled to learn the semantic visual cues (e.g., pieces of chicken, fried eggs, and vegetable leaves) identified by the deep network in the input images. Additionally, we use an image pool consisting of both the original input and the augmented images generated by the attention mechanism. This pool allows the network to learn from a broader set of features, thereby improving the model’s ability to capture diverse and detailed information in the image. At the same time, while low-level features are forwarded to the deep network, many details are lost due to their abstract nature. The attention regions identified by the shallow network assist the deep network in learning essential visual cues (e.g., food textures). To further enhance the training process, we implemented data augmentation techniques to simulate real-world variations in food images. These included random cropping to replicate perspective changes, random horizontal flipping to increase image diversity, and color jittering to account for lighting variations. These augmentations were applied uniformly across all training images to ensure robustness and minimize overfitting. The specific implementation involved resizing images to 448 × 448 pixels before applying these augmentations using PyTorch’s transforms library.

In the $N+1_{st}$ step, we train all network layers and concatenate them with the overall attention regions to extract more refined features. The cross-entropy loss function is employed in all the mentioned stages. During the last phase of MAF-Net training, backpropagation involves a specific approach for calculating the loss function, as follows:

(14) $$L=\alpha L_{Con}+\beta L_{KL}+\gamma L_{SC},$$

where α, β, and γ are balance parameters, and $L_{SC}$ is a new loss function called Subclass Center Loss (SCloss), which we innovatively introduced in MAF-Net. During training, the loss function employed in backpropagation integrates cross-entropy loss to optimize inter-class separability and SCloss to minimize intra-class feature distribution discrepancies. This combination enhances the model’s generalization capability and improves its fine-grained classification performance. The Softmax-based multi-class logistic regression classifier is commonly used in fine-grained image classification. Given an image x, the output prediction probability vector $p(x)=[p_{1}(x),p_{2}(x),\ldots ,p_{C}(x)]$ is computed by the network above. Therefore, the $L_{CE}$ loss can be defined as

(15) $$L_{CE}=-\sum_{i=1}^{C}y_{i}\log(p_{i}(x)),$$

where $y_{i}$ is the indicator variable for the true class label; when the image belongs to class i, $y_{i}=1$, otherwise $y_{i}=0$. However, the Softmax classifier focuses on inter-class separability and ignores intra-class variations. In fine-grained image recognition, especially in food recognition tasks, this deficiency causes features of the same food class to scatter in the feature space and reduces classification performance. To overcome this issue, researchers proposed center loss to compress intra-class variations in fine-grained image recognition tasks [49]. The concept of center loss involves the generation of a centroid vector for each class to represent the class center and measure the distance between this centroid vector and intra-class features in order to minimize feature spread. Expanding on this idea, we propose Subclass Center Loss (SCloss), which is an innovative loss function introduced in MAF-Net. Unlike center loss, which generates a separate center vector for each class and minimizes the distance between the class center and intra-class features, SCloss does not rely on introducing separate center vectors. Instead, it calculates the class center using multiple samples from the same class within a batch, avoiding the challenges related to center initialization and the difficulty of compressing all intra-class samples into a single center when multiple centers exist. Specifically, we create a batch of examples ${\left\{x_{i}\right\}}_{i=1}^{B}$, choose N classes, and then select M examples for each class. Random sampling yields B=N×M examples. For each class in a batch, based on M examples, we first calculate the class center ${\bar{g}}_{c}$, as follows:

(16) $${\bar{g}}_{c}=\frac{1}{M}\sum_{j=1}^{M}g(x_{j};\theta ),$$

where M is the number of samples in class c, and $g(x_{j};\theta )$ is the feature vector of sample $x_{j}$. Then, define SC loss as

(17) $$L_{SC}=\frac{1}{B}\sum_{c=1}^{N}\sum_{j=1}^{M}\left[1-\cos\left(g(x_{j};\theta ),\;{\bar{g}}_{c}\right)\right],$$

where B is the batch size and cos(⋅) is the cosine similarity. The main goal of calculating SCloss is to achieve label smoothing and reduce feature distribution differences within the same class. Unlike center loss, which is sensitive to initialization and faces challenges when multiple class centers exist, SCloss directly calculates the class center from multiple samples within the batch, addressing these challenges and improving classification accuracy. By reducing the intra-class feature dispersion, SCloss significantly enhances the model’s ability to generalize and maintain high accuracy when facing new data.

During training, shallow and deep tasks compete to a certain degree, leading us to gradually optimize these tasks. Each optimization involves different somewhat competing tasks and inputs, training various network components. Thus, completing all tasks outlined in the algorithm is an iterative process. This inference strategy enhances the classification accuracy of the trained model for two reasons: (1) prediction parameters from different network layers and overall prediction parameters offer complementary information and (2) information gained from the original input and overall attention regions provides additional support.
**Algorithm 1.** Training and feature fusion for MAF-Net**Requirement**: Given a dataset $D{=\{(\mathrm{input}}^{i}{,\mathrm{target}}^{i}{)\}}_{i=1}^{I}$(I is the total number of batches in D)1:**for** epoch = 1 to number_of_epochs **do**2:  **for** (input, target) in D **do**3:    
$x_{N},\{x_{1}^{\mathrm{att}},x_{2}^{\mathrm{att}},\ldots ,x_{n}^{\mathrm{att}},\ldots ,x_{N}^{\mathrm{att}},x_{\mathrm{Con}}^{\mathrm{att}}\}\leftarrow \mathrm{input}$
4:    
$L_{N}\leftarrow L_{CE}(f_{N}^{a},\mathrm{target})$
5:    **BACKWARD** ($L_{N}$)6:    **for** n = 1 to N−1 **do**7:      
$\begin{array}{l} \mathrm{input}\leftarrow \mathrm{Randomly}\;\mathrm{select}\;\mathrm{one}\;\mathrm{from}\;\\ \;\;\;\{\mathrm{input}\;,x_{1}^{\mathrm{att}},x_{2}^{\mathrm{att}},\ldots ,x_{n-1}^{\mathrm{att}},x_{n+1}^{\mathrm{att}},\ldots ,x_{N}^{\mathrm{att}}\} \end{array}$
8:      
$L_{n}\leftarrow L_{CE}(f_{n}^{a},\mathrm{target})$
9:      **BACKWARD** ($L_{n}$)10:    
**end for**
11:    
$\begin{array}{l} L_{\mathrm{att}}\leftarrow L_{CE}((f_{1}^{\mathrm{att}}{)}^{a},\mathrm{target})+L_{CE}((f_{2}^{\mathrm{att}}{)}^{a},\mathrm{target})+\ldots +\\ \;\;\;L_{CE}((f_{1}^{\mathrm{att}}{)}^{a},\mathrm{target})+L_{CE}((f_{Con}^{\mathrm{att}}{)}^{a},\mathrm{target})/2 \end{array}$
12:    **BACKWARD** ($L_{\mathrm{att}}$)13:    
$L\leftarrow \alpha L_{Con}+\beta L_{KL}+\gamma L_{SC}$
14:    **BACKWARD** (L)15:  
**end for**
16:**end for**

## 4. Experiment

### 4.1. Dataset

To validate the proposed MAF-Net’s effectiveness, we evaluated our model on two widely used food datasets: CETH Food-101 [47], Vireo Food-172 [24], and UEC FOOD-100 [50].

ETH Food-101 is a Western food dataset comprising 101 food categories, each containing 1000 images, for a total of 101,000 images. Each category is divided into 750 training images and 250 testing images, maintaining a ratio of 3:1. The images were sourced from foodspotting.com, a platform where users upload photographs of the food they consume, typically captured in real-world settings rather than controlled environments. This dataset was developed to advance food recognition technologies, particularly for applications such as mobile food photography, calorie tracking for patients, and enhancing the accessibility of online food photo repositories. It aims to support research in food dish recognition within the field of computer vision, especially considering the high intra-class variability in food images. ETH Food-101 remains unprocessed, allowing researchers to apply operations such as cropping, scaling, and normalization as required. This dataset is widely used for image classification, feature extraction, and transfer learning, serving as a reliable benchmark for fine-grained food image classification tasks.

Vireo Food-172 is a comprehensive Chinese food dataset that encompasses 172 types of Asian dishes and a total of 110,241 images. Developed by researchers at the City University of Hong Kong, this dataset primarily targets ingredient recognition and recipe retrieval, with a specific focus on Chinese cuisine. It includes 172 food categories and 353 ingredient labels, divided into training, validation, and test sets in a ratio of 6:1:3. The data were collected using Chinese food category names as keywords to crawl relevant images from Baidu and Google search engines, followed by rigorous manual screening and verification to ensure image quality. Created to advance food recognition research, particularly in ingredient recognition—a less explored area compared to traditional food classification tasks—this dataset aims to address the zero-shot retrieval problem, enabling the retrieval of recipes for previously unseen food categories based on images. Vireo Food-172 reflects real-world data distribution, featuring an imbalance in the number of images per category, and provides a rich diverse set of visual information captured from various angles and backgrounds, making it an ideal resource for training and evaluating deep learning models in food recognition.

UEC Food-100 is a Japanese food dataset comprising 100 food categories and a total of 12,740 images. Developed in 2012 by researchers at the University of Electro-Communications in Japan, this dataset is primarily utilized for food classification and detection tasks, particularly excelling in identifying multiple food items within single images. The dataset includes both individual food images and composite images containing multiple food items, with varying numbers of images per category. Images were collected via web search engines (such as Google and Baidu) using relevant keywords, followed by rigorous manual screening and verification to ensure high-quality data. The UEC Food-100 dataset aims to advance research in food identification, especially in scenarios involving images with multiple food items, thereby enhancing the accuracy of food region detection and classification.

### 4.2. Experimental Settings

In this study’s experiments, we used ImageNet pre-trained weight initialization parameters as a basis for training all networks. We used the stochastic gradient descent (SGD) algorithm for model training, with 200 iterations, momentum set to 0.9, weight decay set to $5\times 10^{\text{-}4}$, and threshold set to 0.5. The batch size was set to 32, and the learning rate was set to 0.002, adjusted according to the cosine annealing algorithm. In our approach, input images were uniformly resized to a fixed dimension and randomly cropped to the necessary size, accommodating the varying resolutions of the original food image dataset. During the training phase, we employed data augmentation techniques, including random cropping, random horizontal flipping, and color jittering, to enhance model robustness and generalization. Random cropping was chosen to simulate perspective variations by cropping different regions of resized input images (448 × 448 pixels), ensuring robustness to viewpoint changes. Random horizontal flipping, applied with a probability of 0.5, increased data diversity, particularly benefiting symmetrical food images, such as dishes on plates. To address lighting variations common in food photography, color jittering was utilized to adjust brightness, contrast, and saturation within ranges of [0.8, 1.2]. These augmentation techniques are essential in fine-grained image recognition tasks to manage inter-class similarity and intra-class variability, significant challenges in food recognition. For the evaluation phase, test images were resized to a fixed size and center-cropped to maintain consistency with the training process.

The evaluation metrics chosen for this study were Top-1 Accuracy and Top-5 Accuracy, while the PyTorch framework was utilized to develop all neural network models.

### 4.3. Performance Comparison with Baselines

To assess the efficacy of our approach in food category recognition, we performed a comparative analysis between our proposed model and state-of-the-art techniques using the ETH Food-101, Vireo Food-172, and UEC FOOD-100 datasets. The performance metrics on the ETH Food-101 and Vireo Food-172 datasets are summarized in Table 1, whereas the results for the UEC FOOD-100 dataset are detailed in Table 2.

On the ETH Food-101 dataset, our method demonstrates strong performance in both Top-1 and Top-5 classification accuracy under the same experimental setup. Compared to the ResNet50 backbone network, our model improves Top-1 and Top-5 classification accuracy by 2.80% and 0.75%, respectively. However, in terms of Top-1 accuracy, our approach slightly lags behind PRENet using the SENet154 backbone [56] and VOLO-D3 with the Vision Transformer (ViT) backbone [60]. For Top-5 accuracy, our model ranks second only to PRENet with the SENet154 backbone. Notably, despite PRENet’s multilevel feature learning, our proposed method outperforms it by 0.31% and 0.11% in Top-1 and Top-5 accuracy, respectively, when both models use the ResNet50 backbone. The superior performance of PRENet with the SENet154 backbone can be attributed to the SE module in SENet154, which adaptively recalibrates channel features, thereby enhancing the model’s representational and generalization capabilities. However, this complexity also increases the network’s resource requirements. In terms of Top-1 accuracy, VOLO-D3 with the ViT backbone surpasses our model, largely due to ViT’s advantage in capturing long-range dependencies and complex patterns, particularly in global image understanding. In comparison to ViTs, MAF-Net excels in fine-grained food image recognition by enhancing local features through a self-attention mechanism. For instance, on the Vireo Food-172 dataset, MAF-Net outperformed VOLO-D3.

Meanwhile, while some fine-grained recognition methods outperform the baseline network, their performance on food datasets does not match that of standard fine-grained datasets. For instance, DCL [35] performed worse on the ETH Food-101 dataset compared to other fine-grained datasets, possibly because it fails to account for both the texture information of shallow networks and the differences in feature distribution within the same category. Additionally, some fine-grained methods, such as PMG [52], performed even worse than the baseline network. This may be due to PMG not considering the relationships among local features at the same network level, nor promoting the learning of distinct features across different levels during feature extraction. As a result, the model might focus on similar common semantic elements, such as beef in beef noodles. However, certain food groups have flexible compositions and lack consistent semantic characteristics. These experimental results suggest that pre-existing fine-grained techniques may not be the most effective for food item recognition, highlighting that our model is better suited for precise recognition in the domain of cuisine.

On the Vireo Food-172 dataset, our method outperformed most food recognition methods in Top-1 classification accuracy, second only to SGLANet [55]. SGLANet employed two attention mechanisms, one for global feature extraction and the other for local feature extraction, which gave it an advantage in recognition performance. However, the model exhibited a high level of complexity and required extensive training time, rendering it impractical for resource-constrained environments. In Top-5 classification accuracy, our model exhibited better recognition performance than the state-of-the-art, improving by 0.42% and 0.15%, respectively, over the representative CNN method SENet-154 [19] and Transformer method Swin-B [58]. Compared to multi-task methods, our method also performed excellently, surpassing SGLANet by 0.16%. It is worth noting that our method was slightly lower than SGLANet in Top-1 classification accuracy, possibly because SGLANet used a more advanced backbone network, which was SENet154. What is more, the Vireo Food-172 dataset has an issue of uneven category distribution. Due to the limited size of the sample, models such as Swin-B [58] and DAT [59] failed to acquire sufficient features from them, consequently leading to suboptimal performance in these specific categories. Nonetheless, our method demonstrated strong robustness in handling data imbalance and outperformed most existing methods in overall recognition effectiveness.

On the UEC FOOD-100 dataset, our method achieves leading accuracy, outperforming the Ensemble method by 0.59% in Top-1 classification accuracy. This improvement is primarily attributed to the fact that while the Ensemble method [63] predominantly relies on deep global feature extraction and overlooks the significance of shallow local features, it also fails to effectively leverage the self-attention mechanism to dynamically highlight key areas in images, potentially missing fine-grained details. Additionally, the Ensemble method struggles with handling intra-class variations and differences and is less effective in reducing intra-class feature distribution disparities. Consequently, our approach significantly enhances classification performance by placing greater emphasis on local features and accurately identifying critical regions.

### 4.4. Ablation Study

In this section, we conduct ablation experiments to assess the effectiveness of each module in MAF-Net and its impact on model performance. We explore different settings for key parameters, such as self-attention mechanisms, attention region thresholds, feature fusion at various network stages, and the influence of loss function balance parameters on model performance. These experiments aim to validate the robustness of each design and configuration.

Contribution of Self-attention Mechanism: The incorporation of the self-attention mechanism enhances feature representation by generating attention maps at multiple hierarchical levels within the backbone network, thereby augmenting the quality of feature vectors. We fused the feature vectors with attention mechanisms applied to the original input and randomly selected inputs from a pool of original input images and attention areas during subsequent training to achieve data augmentation. To validate the contribution of the self-attention mechanism (SM) and data augmentation (DA), we also compared these with the basic ResNet50. We used the features from the last three layers without using SM and DA for recognition, which we called Simple Feature Fusion (SFF). Throughout the training process, the number of steps, step sequence, and the specific parts of the model trained at each step aligned with those used in MAF-Net. As shown in Table 3, we can observe that (1) Introducing the SM based on SFF improves Top-1 classification accuracy by 2.36% and 3.23%, respectively, on the two datasets and (2) additionally adopting the DA strategy further improves performance, which indicates that fusing feature vectors with attention mechanisms with the original input for data augmentation is effective. We also compared the classification accuracy of feature extraction using feature maps generated by the last three stages of ResNet50, which showed that as the methods improved, the accuracy of different methods at the same stage also exhibited an upward trend.

Influence of threshold θ: After generating the attention map, we use the threshold θ to determine the attention regions. Therefore, the choice of threshold θ has a significant impact on the generation of attention regions. Table 4 shows the classification accuracy performance on the two datasets under different threshold θ values. Experiments showed that setting θ to 0.5 generally provided stable and reliable results; and setting θ too low or too high significantly reduced accuracy. The reason may be that when it is set too low, the attention regions become too broad, including too much irrelevant background information, which increases noise and reduces classification accuracy. When set too high, the attention regions become too focused, probably causing some critical information to be ignored, which affects classification performance. Therefore, in the rest of this paper, we set θ=0.5 by default.

**Figure 3:**
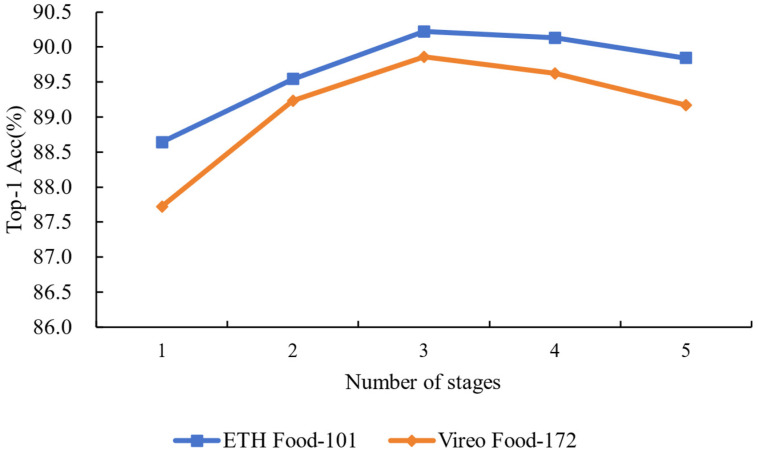
Comparisons of the impact of different stages in selecting the backbone network on experimental results show that the features from the last three stages perform the best.

Influence of the number of stages: We compared the impact of feature fusion with different numbers of stages on model performance to evaluate the effectiveness of the MAF-Net. Specifically, we used ResNet50 as the base network and conducted experiments with the last one, two, three, four, and five stages, respectively. As shown in Figure 3, the model using features from the last three stages performs the best on the ETH Food-101 and Vireo Food-172 datasets. In contrast, the model using only the features from the last stage exhibits lower accuracy, primarily due to the absence of multi-level feature support. Although the model that fuses features from the last two stages shows improved performance; it still does not fully utilize more levels of feature information. When the last four stages are all involved, the model’s accuracy is 90.13% and 89.62%, but the gain is limited and the model complexity significantly increases. Using the features from all five stages significantly increases the training time and computational resource consumption. These results indicate that fusing the features of the last three stages achieves the best balance between performance and computational complexity.

**Figure 4:**
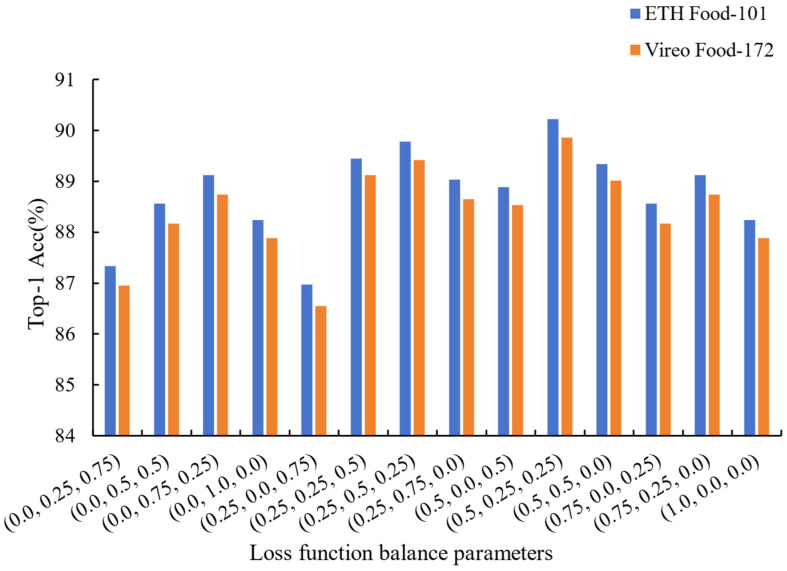
Comparisons of the effects of using different balancing parameters on experimental results indicate that the performance is poor when using only one or two of the loss functions $L_{Con}$, $L_{SC}$, or $L_{KL}$, while the highest recognition accuracy is achieved with the balancing parameters set to (0.5, 0.25, 0.25).

Influence of loss function balance parameters: our model used three loss functions, namely $L_{Con}$, $L_{SC}$, and $L_{KL}$, during the final backpropagation process. We evaluated the impact of different weight settings on model performance by weighted summation of these three loss functions. As shown in Figure 4, the model attains the highest performance on both datasets when the loss function weights are configured to (0.5, 0.25, 0.25). When only one of the loss functions is considered and the others are set to 0, the model’s performance significantly drops. This phenomenon indicates that a single loss function cannot adequately capture and optimize the various features and classification boundaries of the model. Additionally, we can observe that when considering only two combinations of loss functions, the results are comparatively inferior to those obtained by assigning weights of (0.5, 0.25, 0.25) to the loss functions. The attention mechanism loss function mainly focuses on feature fusion, $L_{KL}$ on distribution differences, and $L_{SC}$ on enhancing class center discrimination. The combination of these three provides more comprehensive and balanced optimization, which significantly improves the overall performance of the model.

### 4.5. Visualization Analysis

**Figure 5:**
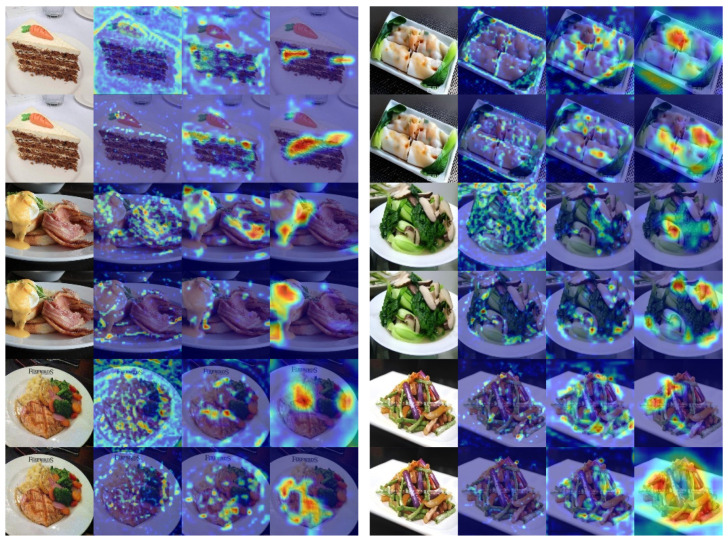
Visualization results of MAF-Net on some samples from CETH Food-101 (**left**) and Vireo Food-172 (**right**). From left to right are the input images, Stage-3, Stage-4, and Stage-5, with rows 1, 3, and 5 showing the original network, and rows 2, 4, and 6 showing MAF-Net.

To enhance the investigation of our approach’s efficacy, we employ GradCAM for visual examination [64]. Figure 5 presents the output feature maps at different learning stages and compares them with those generated by the original network to encompass a broader range of information. The original network relies solely on the feature maps from the last three layers of the ResNet50 network, lacking both a self-attention mechanism and KL-divergence optimization. Our method shows broader attention regions in the following examples and captures more detailed information as the training stages progress. Especially in different stages with KL-divergence optimization, our method effectively captures various local features. For example, with “Rice Noodles” (first row on the right), compared to the original network Stage-5 (second row on the right), which can only recognize “Rice Noodles”, our network can also recognize “Green Vegetables” at Stage-5. At the same time, MAF-Net focuses on the bottom right features of “Rice Noodles” in Stage-5. In contrast, in Stage-4, it focuses on the top right and bottom left features, thanks to KL-divergence optimization allowing the network to focus on more diverse feature areas. Additionally, the visualization results of our network in Stage-3 show that it has a good recognition effect on food texture features, significantly aiding food recognition. In summary, our proposed method significantly enhances the model’s performance in food image recognition. This multi-stage learning process and optimization strategy enable our network to more comprehensively understand and recognize food features when dealing with complex images and demonstrate superior performance.

## 5. Conclusions and Discussion

In this study, we introduce a novel architecture, MAF-Net, for food image recognition. MAF-Net enhances local feature representation by integrating features from various levels of the CNN backbone through a self-attention mechanism. This approach improves data augmentation by combining attention-enhanced feature vectors with the original input. Additionally, we maximize the KL-divergence across different stages, encouraging the model to focus on distinct local regions and capture finer details. Our key innovation is the introduction of SCloss, which facilitates label smoothing, reduces intra-class feature distribution disparities, and enhances generalization. MAF-Net achieves comprehensive feature enhancement across multiple levels, within and between layers, and across entire classes. Experimental results demonstrate that MAF-Net effectively addresses the target problem and significantly improves the accuracy of food image classification tasks. While the multi-step training strategy increases training time compared to the original backbone, the inference cost is more critical for practical applications. Future work will focus on enhancing training efficiency without compromising model accuracy.

Despite its strong performance in food image recognition, MAF-Net has some limitations that warrant further investigation. The multi-step training strategy introduces additional computational overhead, which could be a concern in resource-constrained environments. Additionally, our evaluation, while comprehensive across three datasets—CETH Food-101, Vireo Food-172, and UEC Food-100—leaves room to explore its generalizability to other fine-grained classification domains. Future research will focus on optimizing the training process, exploring lightweight architectures, and testing MAF-Net on a broader range of datasets to enhance its robustness and adaptability.

## Figures and Tables

**Table 1:** Comparison of different FOOD Recognition methods on ETH Food-101 and VIREO Food-172 datasets (%).

Method	Backbone	ETH Food-101		Vireo Food-172	
		Top-1 Acc	Top-5 Acc	Top-1 Acc	Top-5 Acc
ResNet152 + SVM-RBF [21]	ResNet152	64.98	-	-	-
FS_UAMS [7]	Inceptionv3	-	-	89.26	-
ResNet50 [25]	ResNet50	87.42	97.40	-	-
DenseNet161 [51]	DenseNet16	-	-	86.98	97.31
SENet-154 [19]	ResNeXt-50	88.68	97.62	88.78	97.76
PAR-Net [12]	ResNet101	89.30	-	89.60	-
DCL [35]	ResNet50	88.90	97.82	-	-
PMG [52]	ResNet50	86.93	97.21	-	-
WS-DAN [53]	Inceptionv3	88.90	98.11	-	-
NTS-NET [54]	ResNet50	89.40	97.80	-	-
HBP [55]	ResNet50	86.23	97.13	-	-
PRENet [56]	ResNet50	89.91	98.04	-	-
PRENet [56]	SENet154	**90.74**	**98.48**	-	-
SGLANet [57]	SENet154	89.69	98.01	**90.30**	98.03
Swin-B [58]	Transformer	89.78	97.98	89.15	98.02
DAT [59]	Transformer	90.04	98.12	89.25	98.12
VOLO-D3 [60]	ViT	90.53	98.07	89.72	97.86
MAF-Net	ResNet50	90.22	98.15	89.86	**98.18**

**Table 2:** Comparison of different FOOD Recognition methods on UEC FOOD-100 (%).

Method	Backbone	Top-1 Acc	Top-5 Acc
SELC [61]	ELM	84.30	-
WARN [62]	Wide Residual Networks (WRN)	88.50	-
Ensemble [63]	ResNeXt and DenseNet	90.02	-
MAF-net	ResNet50	**90.61**	**98.93**

**Table 3:** Ablation studies on the self-attention mechanism and data augmentation (%).

	ETH Food-101				Vireo Food-172			
	p_1_	p_2_	p_3_	Top-1	p_1_	p_2_	p_3_	Top-1
SFF	86.43	87.23	86.79	87.86	82.87	86.12	85.72	86.63
SFF + SM	87.59	89.17	88.94	89.76	86.14	88.92	88.76	89.23
SFF + SM + DA	88.13	89.67	89.21	90.22	86.53	89.52	89.83	89.86

**Table 4:** Ablation studies on different threshold (θ) values (%).

θ	0.1	0.3	0.5	0.7	0.9
ETH Food-101	88.97	89.45	90.22	89.63	89.07
Vireo Food-172	87.68	89.23	89.86	89.06	87.54

## Data Availability

The experimental data used in this study are sourced from several publicly available datasets, including ETH Food-101 (accessible at: https://data.vision.ee.ethz.ch/cvl/datasets_extra/food-101/), accessed on 30 January 2025; Vireo Food-172 (accessible at: https://fvl.fudan.edu.cn/dataset/vireofood172/list.htm), accessed on 30 January 2025; and UEC-Food100 (accessible at: http://foodcam.mobi/dataset100.html), accessed on 30 January 2025. We sincerely appreciate the provision of these resources, which have significantly contributed to the conduct of this research.
